# Shotgun metagenomics reveals a wide array of antibiotic resistance genes and mobile elements in a polluted lake in India

**DOI:** 10.3389/fmicb.2014.00648

**Published:** 2014-12-02

**Authors:** Johan Bengtsson-Palme, Fredrik Boulund, Jerker Fick, Erik Kristiansson, D. G. Joakim Larsson

**Affiliations:** ^1^Department of Infectious Diseases, Institute of Biomedicine, The Sahlgrenska Academy, University of GothenburgGothenburg, Sweden; ^2^Department of Mathematical Sciences, Chalmers University of Technology and University of GothenburgGothenburg, Sweden; ^3^Department of Chemistry, Umeå UniversityUmeå, Sweden

**Keywords:** antibiotic resistance, horizontal gene transfer, lake sediment, metagenomics, mobile genetic elements, pharmaceutical pollution, plasmids

## Abstract

There is increasing evidence for an environmental origin of many antibiotic resistance genes. Consequently, it is important to identify environments of particular risk for selecting and maintaining such resistance factors. In this study, we described the diversity of antibiotic resistance genes in an Indian lake subjected to industrial pollution with fluoroquinolone antibiotics. We also assessed the genetic context of the identified resistance genes, to try to predict their genetic transferability. The lake harbored a wide range of resistance genes (81 identified gene types) against essentially every major class of antibiotics, as well as genes responsible for mobilization of genetic material. Resistance genes were estimated to be 7000 times more abundant than in a Swedish lake included for comparison, where only eight resistance genes were found. The *sul2* and *qnrD* genes were the most common resistance genes in the Indian lake. Twenty-six known and 21 putative novel plasmids were recovered in the Indian lake metagenome, which, together with the genes found, indicate a large potential for horizontal gene transfer through conjugation. Interestingly, the microbial community of the lake still included a wide range of taxa, suggesting that, across most phyla, bacteria has adapted relatively well to this highly polluted environment. Based on the wide range and high abundance of known resistance factors we have detected, it is plausible that yet unrecognized resistance genes are also present in the lake. Thus, we conclude that environments polluted with waste from antibiotic manufacturing could be important reservoirs for mobile antibiotic resistance genes.

## Introduction

Antibiotics constitute our core line of defense against infectious diseases caused by bacteria and are also fundamental for our ability to perform advanced surgery and treat cancer. However, in later years, an antibiotic resistance crisis has emerged, causing increased mortalities and substantial costs; expenses have recently been estimated to be over 1.5 billion euros every year in Europe alone (Norrby et al., 2009). There is increasing evidence that the resistance we see in pathogens did not initially appear in the clinical setting, but that environmental bacteria have contributed to the resistance gene pool shared among pathogens today (Martinez, 2008; Forsberg et al., 2012; Gaze et al., 2013; Wellington et al., 2013). Resistance genes are commonly encountered on, or associated with, mobile genetic elements (Stokes and Gillings, 2011; Heuer and Smalla, 2012) such as plasmids (Schlüter et al., 2007; Szczepanowski et al., 2008), integrons (Gaze et al., 2011), and transposons (Toleman and Walsh, 2011; Nigro and Hall, 2012). This enables their transfer within and between bacterial cells and species, and their genetic context should therefore be considered a major factor when assessing the risk of transfer from a source environment, and onwards into clinically relevant bacteria. Even if much evidence points toward an ancient origin for most—if not all—mobile resistance determinants (D'Costa et al., 2011), the picture of the environments in which they are initially selected for, as well as their dissemination routes from there into human pathogens, is much less clear. It is reasonable to assume that such transfer of genes from the environment will occur in the future, and that we can expect pathogens to pick up additional resistance determinants from the environmental resistome. To mitigate this problem, it is important to identify source environments where resistance genes can be selected for, and subsequently transferred into human or animal pathogens. Likely, environments under sufficient selection pressure to maintain a range of known antibiotic resistance genes in relatively large quantities also harbor a fair number of yet undescribed resistance factors, and thereby may serve as a recruitment ground for novel resistance determinants. Environments polluted by direct discharges from manufacturing of antibiotics appear to provide such conditions (Larsson et al., 2007; Li et al., 2008a,b; Larsson, 2014).

We have for some years studied the Patancheru area in India, housing a range of pharmaceutical industries. Discharges, both from a common effluent treatment plant and illegal dumping of production waste (Greenpeace, 2004; Boralkar et al., 2005; Larsson et al., 2007), has led to unprecedented antibiotic contamination of both surface and ground water (Fick et al., 2009) as well as river sediment (Kristiansson et al., 2011). In this work, we have investigated another environment where resistance genes may flourish; Kazipally lake, located in the same area, and polluted by direct dumping of waste from pharmaceutical production. Fick et al. (2009) reported several pharmaceuticals in the surface water of Kazipally lake, with concentrations of norfloxacin of up to 520,000 ng/L and enoxacin of up to 84,000 ng/L, approximately corresponding to the clinical breakpoint for fluoroquinolone resistance in enterobacteriacea (European Society of Clinical Microbiology and Infectious Diseases, 2014). To capture a broad diversity of resistance genes, we have here used shotgun sequencing metagenomics to investigate the bacterial community of the lake. In contrast to PCR, this approach allow us to search for in principle any known resistance gene in the same analysis, given that it is present in sufficient abundance to be detected using the sequencing effort of the study. Shotgun metagenomics also offers the opportunity to study the taxonomic and functional content of the bacterial communities in a comprehensive manner. We have previously studied river sediments from the Patancheru area using shotgun pyrosequencing, revealing a high abundance of certain antibiotic resistance genes (Kristiansson et al., 2011). It is clear, however, that the amount of data obtained by this sequencing technique only allows us to scratch the surface of the environmental resistome. To be able to more comprehensively describe the resistome of the polluted lake, we have therefore employed Illumina sequencing, as it delivers considerably more DNA data than does pyrosequencing, given the same sequencing costs.

Using the metagenomic sequencing data we have described the diversity of resistance genes in the highly polluted Kazipally lake, to better understand how the selection pressures here, and possibly in environments similar to this lake, could shape the local resistome. Furthermore, we aimed to define the genetic context of the identified resistance genes, to evaluate their potential to be transferred between bacteria. Several studies have suggested that stressors such as antibiotics could contribute to a general mobilization of genetic material in bacterial communities (Beaber et al., 2004; Prudhomme et al., 2006), and we have therefore specifically looked for genes involved in genetic mobility, and have also identified known and novel plasmids. In addition, strong environmental disturbances have been proposed to reduce the taxonomic diversity of ecological communities (Banks et al., 2013). We have thus explored the diversity of microbial taxa in the lake to understand if such a shift in community structure is likely to have taken place due to the considerable releases of chemicals into the lake.

## Materials and methods

### Sampling and DNA sequencing

Sediment samples were collected from the Kazipally lake in the Patancheru region, close to Hyderabad in India (N 17°34.45, E 078°21.38). We did not have access to sediment from any nearby, non-polluted lake that could serve as a local reference. Although there are many environmental metagenomes available for comparisons (see discussion), we collected sediment samples from the Nydalasjön lake, close to Umeå, Sweden (N 63°49.30, E 020°20.85) and analyzed it in a directly comparable way. The latter is a healthy lake with viable populations of fish, insects and other fauna. It is used for recreational swimming activities in the summer, and it does not receive any direct discharges of sewage or industrial waste. Six samples were collected at the Indian site, and 20 samples at the Swedish site. Samples from within the same lake were pooled in order to minimize spatial variability. For each sample, the top 5 cm of sediment were collected in test tubes. Indian samples were refrigerated for approximately 1 week before shipping to Sweden where they were frozen at −80°C, whereas the Swedish samples were frozen immediately. Although an extended time before freezing might have influenced the community structure to some extent, there are no apparent reasons to believe that this would select for bacteria harboring antibiotic resistance genes in any consistent way. DNA was extracted from the samples using the PowerSoil DNA isolation kit (MO BIO, Carlsbad, CA) according to the manufacturer's instructions. Extracting sufficient amounts of high quality DNA from the polluted Indian sediment turned out to be a challenge, likely due to chemicals in the sediment material interfering with the DNA extraction process, in combination with low DNA concentrations. We solved this by amplifying the extracted DNA using the Repli-G mini kit (Qiagen, Hilden, Germany), according to the manufacturer's instructions. The same treatment was also applied to the sediment samples from the Swedish lake, to enable better comparisons. It should be acknowledged that Repli-G as well as other amplification procedures might not amplify all sequences to the same extent (Pinard et al., 2006), and thus quantitative estimates should be interpreted with some caution. As the samples from both lakes were amplified using Repli-G, potential biases should affect the DNA libraries in the same way. Paired-end sequencing libraries (2 × 100 bp) were prepared using the TrueSeq DNA Kit for multiplexing. Sequencing was done at Science for Life Laboratories (Stockholm, Sweden) using the Illumina HiSeq2000 platform. The sequence data have been submitted to the European Nucleotide Archive under project accession number PRJEB6102 (http://www.ebi.ac.uk/ena/data/view/PRJEB6102).

### Sequence analysis

The quality of each sequencing library was assessed using FastQC (Andrews, 2010), and reads with lower quality scores than 28 over more than 75% of the read were discarded using a paired-end aware read filterer (Pearf, part of the PETKit; Bengtsson-Palme, 2012). In addition, when more than 5% of the bases in a read fell below a quality score of 28, the read was trimmed at the first low-quality base. Reads trimmed to less than 30 bp were discarded. If one read in a read-pair was discarded, the paired read was discarded as well (Pearf options “-q 28 -f 0.25 -t 0.05 -l 30”).

Quality filtered reads were mapped against the Resqu antibiotic resistance database (version 1.1; http://www.1928diagnostics.com/resdb). The database contains 3019 non-redundant protein sequences corresponding to 325 resistance gene families (see Table S1 for the complete list). All data in the database have been manually extracted from the literature. The mapping was done using Vmatch (Kurtz, 2010), allowing one mismatched amino acid per translated read (options “-showdesc 60 -dnavsprot 11 -l 20 -h 1”). The number of matches to each resistance gene were counted, and subsequently normalized to the length of the respective gene. The length-normalized numbers were then further normalized to the number of bacterial 16S rRNA sequences divided by the length of the 16S gene, yielding an approximation of the number of genes per 16S rRNA sequence for each resistance gene, while still avoiding bias due to differential length of the resistance genes.

Read pairs were scanned with Metaxa 2.0 (Bengtsson et al., 2011; Bengtsson-Palme et al., 2014) to extract bacterial 16S rRNA (SSU) sequences (default options). Read pairs identified as bacterial SSUs by Metaxa were assigned to and grafted on their closest matching bacterial rRNA sequences in SILVA (release 108) using Megraft (version 1.0.2; Bengtsson et al., 2012), clustered in Usearch (Edgar, 2010; options “–usersort –id 0.97”), and further subjected to rarefaction analysis in the R package Vegan (Oksanen et al., 2011).

### Metagenomic assembly

Assembly of the quality filtered reads was done using Velvet (Zerbino and Birney, 2008) on the C3SE computer cluster at Chalmers University of Technology. Non-circular contigs were further post-processed in Peacat (part of the PETKit; Bengtsson-Palme, 2012; options: “–overlap 25 -n”) to further merge contigs with overlaps of at least 25 bp (Table S2). Reads were mapped back to contigs using BLAT (Kent, 2002; options “-out=blast8 -q=dna -t=dna -minIdentity=95”). At least 50 matching bp were required for a read to be counted as mapped. Only the best match of each mapped read was used for determination of feature abundance. Annotation was performed using BLAST (Altschul et al., 1997) against plasmids and viruses in GenBank (Benson et al., 2014; blastall options “-p blastn -m 8 -a 16 -e 1e-4 -F F”). Annotation against the NCBI non-redundant protein database was performed using BLAT (options “-out=blast8 -q=prot -t=dnax”). Translated contigs were searched against the Pfam (Finn et al., 2014) and TIGRFAM (Haft et al., 2013) databases and HMM profiles for type 4 secretion systems (Smillie et al., 2010; Guglielmini et al., 2011) using HMMER (Eddy, 2010; hmmsearch options “–noali –cut_ga”). Contigs were annotated for resistance genes using an in-house pipeline for resistance gene and mobile element finding. All annotations were then added to a database containing all annotated features, which was subsequently queried at different, more specific, cutoffs depending on what data that was requested.

Additional assembly of resistance gene contigs where carried out by a custom iterative approach employing Vmatch (Kurtz, 2010) and Trinity (Grabherr et al., 2011). In this approach, reads mapped to a resistance gene were extracted and used as seeds for a Vmatch search against the complete set of read pairs matching with at least 49 bp to any of the seed reads. These reads were then assembled using Trinity. The resulting contigs were then used as seeds for another search using Vmatch to the complete set of quality-filtered reads, as above. All matches (including the previously matching read pairs) were then used for another Trinity run. This iterative process was then repeated until the total number of assembled nucleotides started to drop rather than increase (40 iterations in this case). The script used has been made available as a software package (Bengtsson-Palme, 2013). The complete set of options used were “–cpu 8 –overlap 49 –contig_min 150.”

### Abundance analysis

Abundances of Pfam and TIGRFAM families were estimated by calculating the mean number of reads that could be mapped to each specific region matching a Pfam or TIGRFAM family, such that at least 75 bp of the read was mapped to that genetic region, and then subsequently calculating the sum of all those mean values. This number was then divided by the number of reads that passed quality filtering from that sequencing library, yielding a relative abundance estimate. All abundances were then multiplied by 1 million, and can therefore be interpreted as the number of reads mapped to that feature in every million reads generated (Tables S3, S4). Gene Ontology (GO; Ashburner et al., 2000) terms were mapped to the Pfam families using the Pfam2GO mapping supplied by the GO website, and to TIGRFAM families using the mapping supplied by TIGRFAM. GO terms were then mapped back to the third level in the GO hierarchy to generate comparable data for differently annotated biological processes and molecular functions (Tables S5–S9).

### Identification of plasmids

Known plasmids were identified by mapping the quality-filtered reads from the Indian and Swedish lakes to the NCBI plasmid genomes dataset, using BLAT (options “-out=blast8 -q=dna -t=dna -minIdentity=95”). Plasmids with less than 90% coverage were discarded (and thus not counted). Novel plasmids were identified by identifying contigs longer than 3000 bp, with a circular overlap of at least 30 bp, which also had read pairs that mapped to both ends of the contig. Additionally, we also demanded that each circular contig should contain a relaxase or a conjugation system to be classified as a plasmid, to exclude viruses and other non-plasmid circular DNA elements.

## Results

Using Illumina sequencing, we generated 6.69 Gbp of data, corresponding to 21,201,938 paired-end reads passing quality filtering of sediment samples from the Indian lake and 33,422,609 reads from the Swedish lake (Table 1). The reads were assembled separately using Velvet, producing roughly 400,000 contigs for each lake (Table S2).

**Table 1:** **Summary of the two sequencing libraries used in this study**.

**Sample**	**Raw read pairs**	**Gbp**	**Passed filtering**	**% passed**	**SSU rRNA**	**% SSU rRNA**	**Contigs**	**Total resistance gene reads**	**% resistance genes**
Indian lake sediment	33 466 587	6.69	21 201 938	63.35	7310	0.034	419 550	66 960	0.32
Swedish lake sediment	69 474 306	13.89	33 422 609	48.11	8841	0.026	415 530	10	<0.01

**Figure 1:**
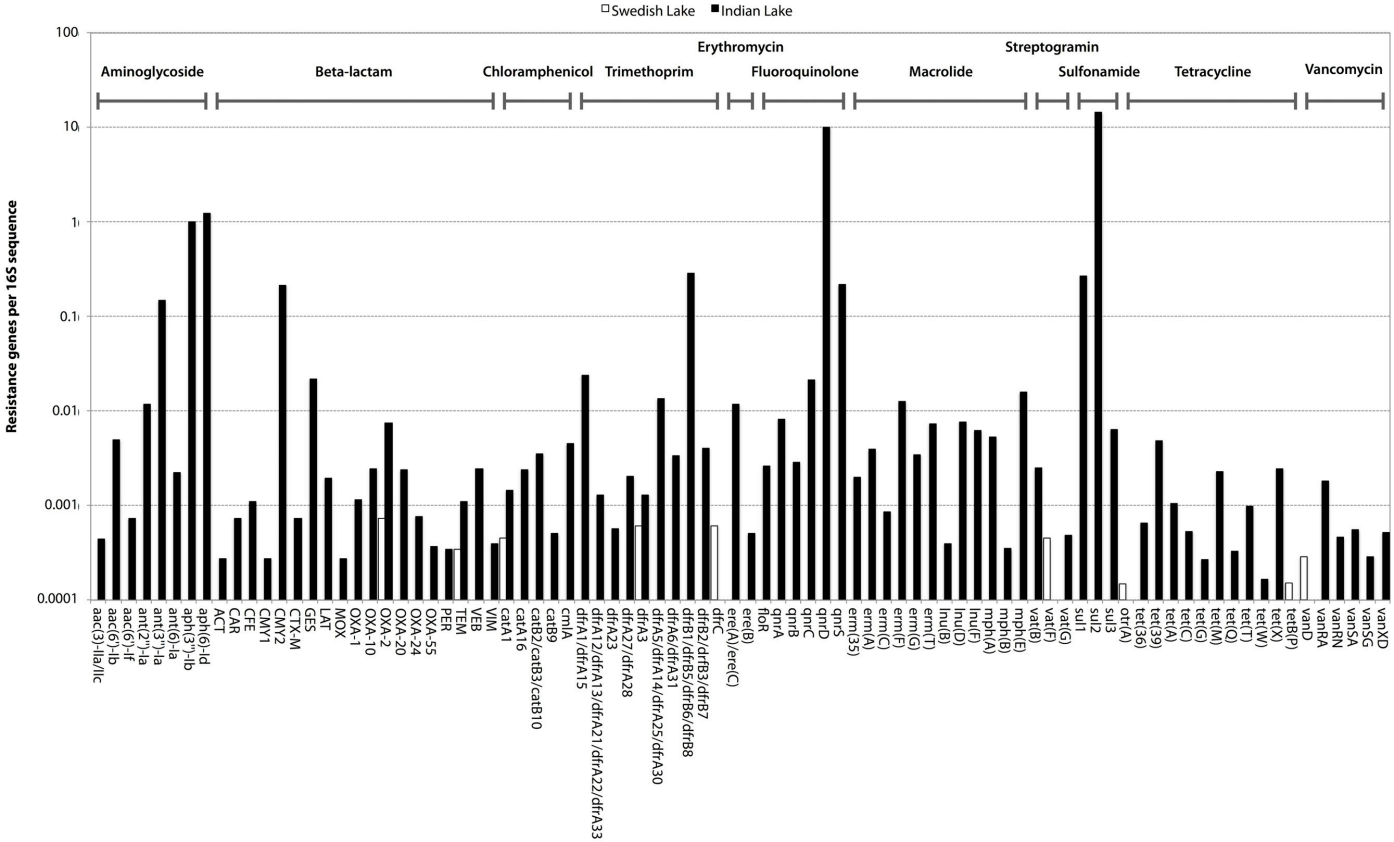
**Abundance of encountered mobile antibiotic resistance genes in the Swedish (white) and Indian lakes (black), normalized to the number of 16S rRNA sequences in the samples, clearly revealing the striking difference in both abundance and diversity of resistance genes**. The scale on the y-axis is logarithmic, and resistance genes are ordered according to class (as indicated at the top of the figure).

In the Indian lake, we found 66,960 reads matching resistance genes, while only 10 reads matched in the Swedish lake metagenome. After normalization to gene length and the number of bacterial 16S sequences in each sample, we found about 28.4 resistance genes per 16S sequence in the Indian sample and 0.004 in the Swedish lake (a more than 7000-fold difference). While a large part of the resistance gene content in the polluted lake can be attributed to the sulfonamide resistance gene *sul2* (51.2% of resistance genes detected) and the quinolone resistance gene *qnrD* (35.8%), 81 types of resistance genes could be detected, conferring resistance to in principle all major classes of antibiotics (Figure 1; Table 2).

**Table 2:** **Top 20 antibiotic resistance genes in the Indian lake**.

**Resistance gene**	**Swedish lake**	**Indian lake**
*sul2*	0	37332
*qnrD*	0	20603
*aph(6)-Id*	0	3221
*aph(3′)-Ib*	0	2595
*CMY2*	0	778
*sul1*	0	713
*qnrS*	0	450
*ant(3′)-Ia*	0	396
*dfrB1/dfrB5/dfrB6/dfrB8*	0	216
*GES*	0	60
*ere(A)/ere(C)*	0	45
*qnrC*	0	45
*mph(E)*	0	44
*dfrA1/dfrA15*	0	36
*erm(F)*	0	32
*ant(2′)-Ia*	0	27
*dfrA5/dfrA14/dfrA25/dfrA30*	0	20
*OXA-2*	2	19
*cmlA*	0	18
*tet(39)*	0	18

**Figure 2:**
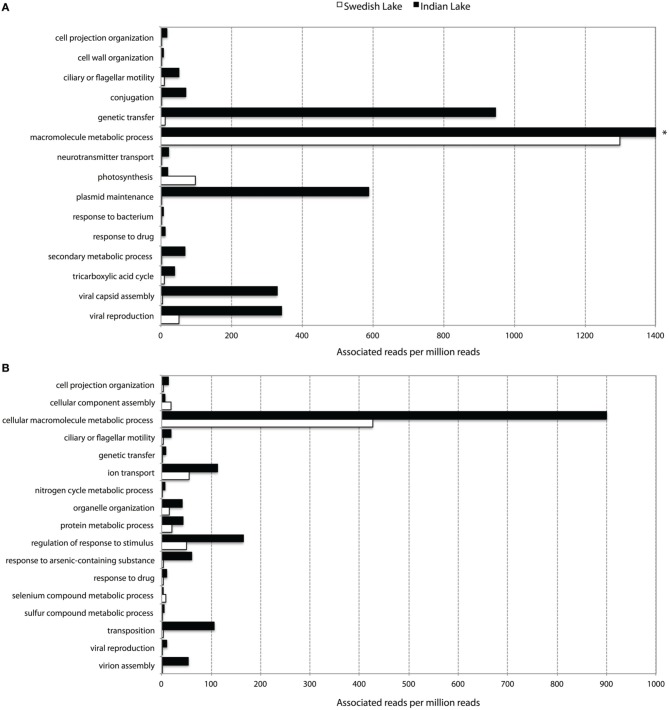
**Abundance of level 3 biological process GO-terms (based on Pfam (A) and TIGRFAM (B) families) with more than two times difference in their prevalence between the Indian (black) and the Swedish (white) lake**. Only terms representing more than five out of a million reads in at least one of the lakes are shown. ^*^The “macromolecular metabolic process” GO-term had 8954.60 occurrences per million reads in the Indian lake, and was cut for viewing purposes.

To further assess key functions of the microbial community in the polluted lake, we annotated the assembled contigs against the Pfam, TIGRFAM, and NCBI non-redundant protein databases. We contrasted this with the corresponding analysis of the non-polluted Swedish lake. While only two samples will not allow any proper statistical support for over- and under-representations of protein families, the normalized fold-change between the Indian and Swedish lake can provide us with protein families and features of outstanding interest in these communities. When considering the Pfam protein families with radically higher abundance in the polluted lake, the most striking features are the large proportion of protein families associated with mobility (Figure 2), and a number of protein families indicating higher abundances of phages. Looking at the most over-represented protein families in the Swedish lake, we instead find many proteins related to photosynthesis, which are almost completely absent in the Indian lake.

**Figure 3:**
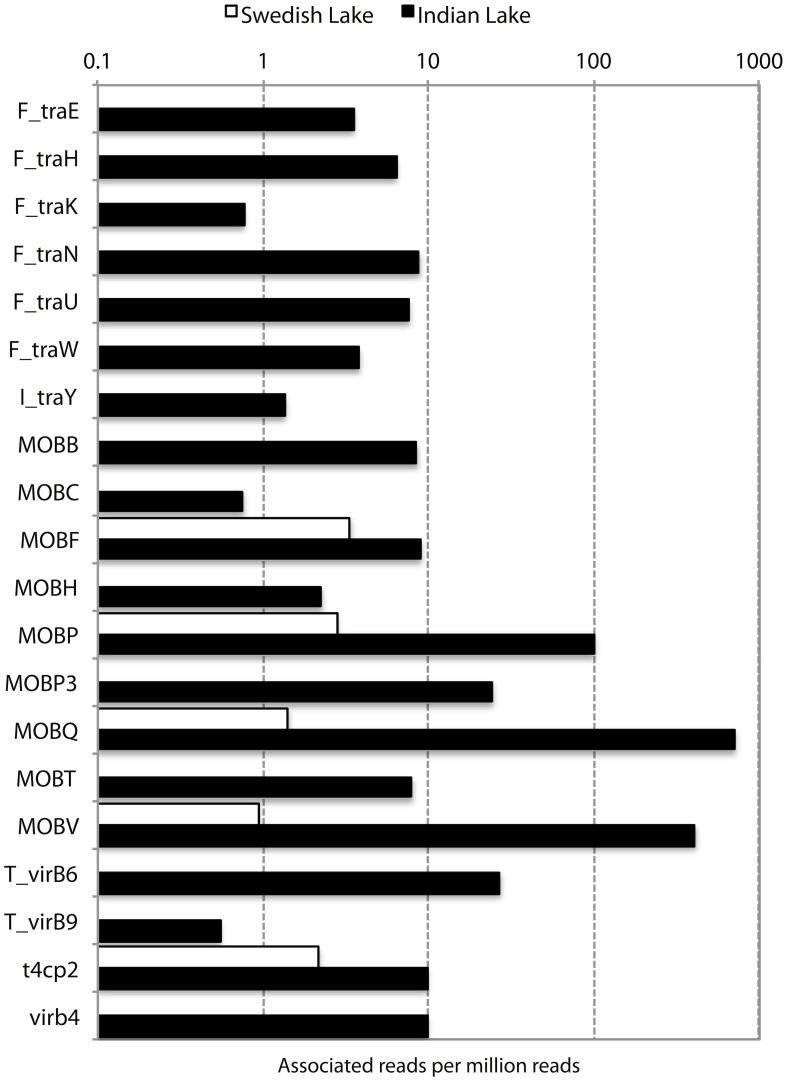
**Abundance of different conjugation systems in the Indian (black) and Swedish (white) lake, in reads per million sequences**.

**Figure 4:**
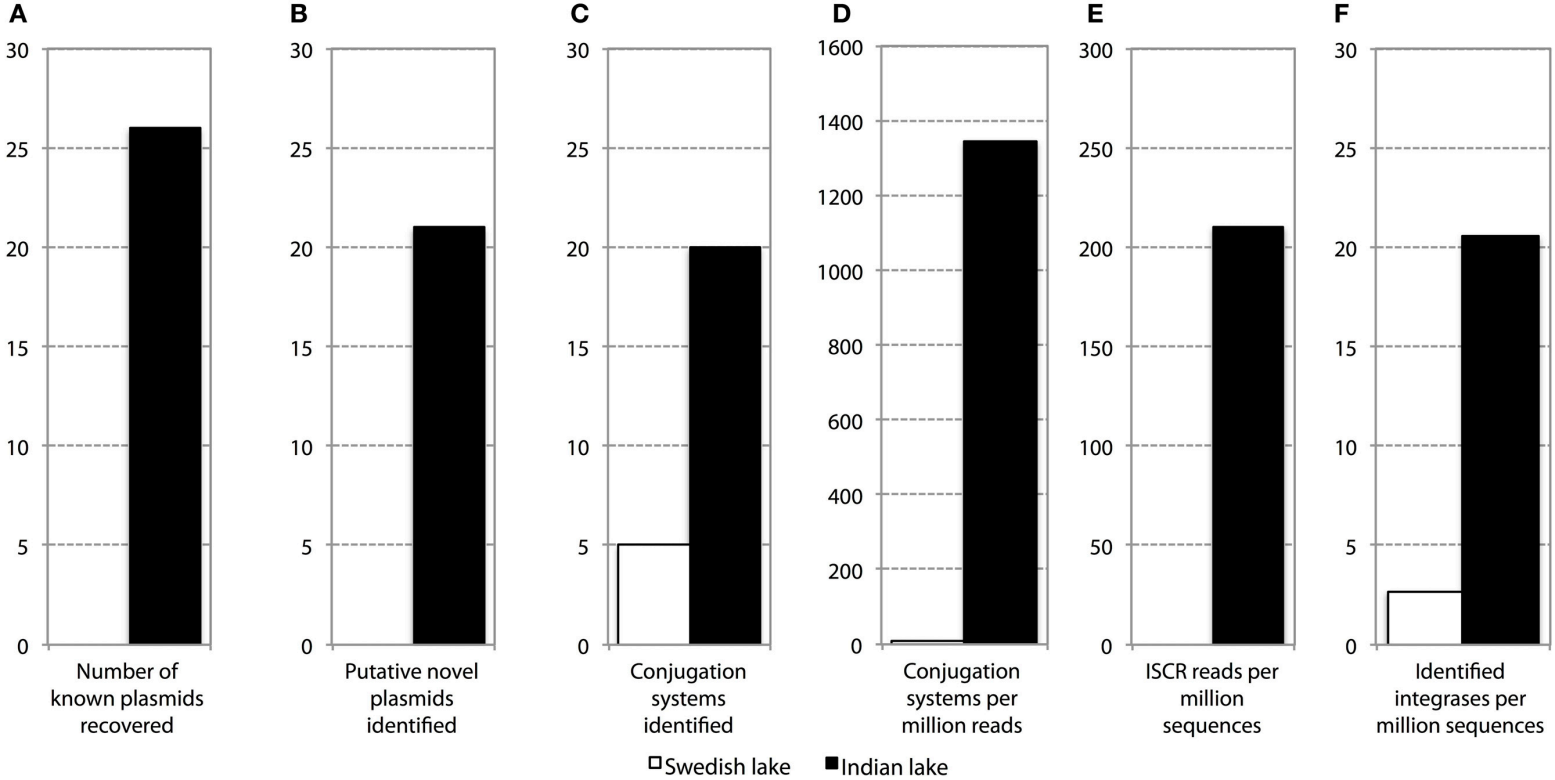
**Abundance and diversity of plasmids and other mobile genetic features in the Swedish (white) and Indian (black) lake. (A)** Number of plasmids in the NCBI plasmid genome database that had more than 90% coverage of reads in the lake datasets. **(B)** Number of hitherto undescribed putative plasmids recovered from in the two lakes. Only circular contigs of at least 3000 bp length with conjugation systems present were considered to be novel plasmids. **(C)** Number of different conjugation systems identified in the two lakes. See also Figure 3. **(D)** Proportion of reads mapping to the conjugation systems in **(C)**. **(E)** Proportion of reads mapping to the ISCR elements. **(F)** Proportion of reads mapping to *intI* integrons.

**Figure 5:**
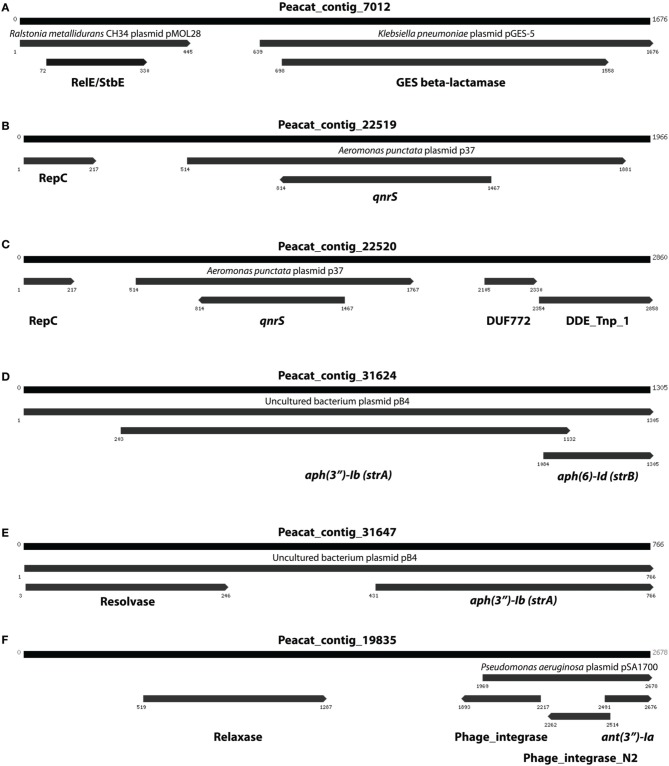
**Examples of assembled contigs containing antibiotic resistance genes from the Indian lake**. **(A)** A RelE/StbE toxin-antitoxin system close to a GES extended spectrum beta-lactamase gene. **(B)** Fluoroquinolone resistance gene *qnrS*, located close to a gene encoding a bacterial plasmid replicase RepC protein. **(C)** An identical *qnrS* gene together with the transposon-associated Pfam domain DUF772 and a DDE transposase domain. **(D)** The streptomycin resistance genes *aph(3″)-Ib* and *aph(6)-Id*. **(E)** The same streptomycin resistance genes in a similar, but not identical, context. **(F)** The aminoglycoside resistance gene *ant(3″)-Ia* together with a gene containing a relaxase domain and a DNA integrase gene typically found in class 1 integrons. Numbers indicate the positions on the contig.

In addition to resistance genes, we also found a wide range of genes enhancing mobility of genetic material in the Indian samples (Figure 3, Figures S1, S2), such as plasmid conjugation systems, integrases, and transposases. Furthermore, we could detect a large number of *intI* integrons and ISCR sequences. The distribution of integrase sequences was distinctly different in the two lakes, with the Indian lake showing higher overall integrase levels (Figure 4), which could primarily be ascribed to ISCR2, *intI1*, and *intI9* (Figure S2). Several of these mobile elements were located in close proximity to resistance genes (Figure 5). For example, we found a *RelE*/*StbE* toxin-antitoxin system close to a GES extended spectrum beta-lactamase gene (Weldhagen, 2006; Figure 5A). We also found the fluoroquinolone resistance gene *qnrS*, located close to a gene encoding a bacterial plasmid replicase *RepC* protein (100% identity to *Escherichia coli* protein WP_001672015.1; Figure 5B). An identical *qnrS* gene was also found in a similar context, but with the transposon-associated Pfam domain DUF772 and a DDE transposase domain located downstream of the *qnrS* gene. This open reading frame had 73% amino acid identity to an *Azoarcus sp*. IS5 transposase (ISAzo41; Siguier et al., 2006), which has not previously been associated with antibiotic resistance genes. However, the *qnrS* gene could be part of this transposable element (Figure 5C). We also found the commonly occurring streptomycin resistance genes *aph(3′)-Ib* (*strA*) and *aph(6)-Id* (*strB*) on a contig with 99.9% similarity to a previously sequenced plasmid (AJ431260.1; Figures 5D,E; Tauch et al., 2003). This pair of genes is, however, often found on mobile elements, including transposons and plasmids (Sundin and Bender, 1996; Sundin, 2002), making their relation to that specific plasmid somewhat dubious. The aminoglycoside resistance gene *ant(3′)-Ia* was found together with a gene containing a relaxase domain with a weak hit to *MobP3* (*E*-value 4.5e-11; Smillie et al., 2010), as well as a DNA integrase gene typically found in class 1 integrons (*intI1*; 100% identity to *Klebsiella pneumoniae* protein ACV33249.1; Figure 5F). The contig did not have full-length coverage of any known plasmid, potentially signifying that this constellation of genes here appear in a new context.

**Figure 6:**
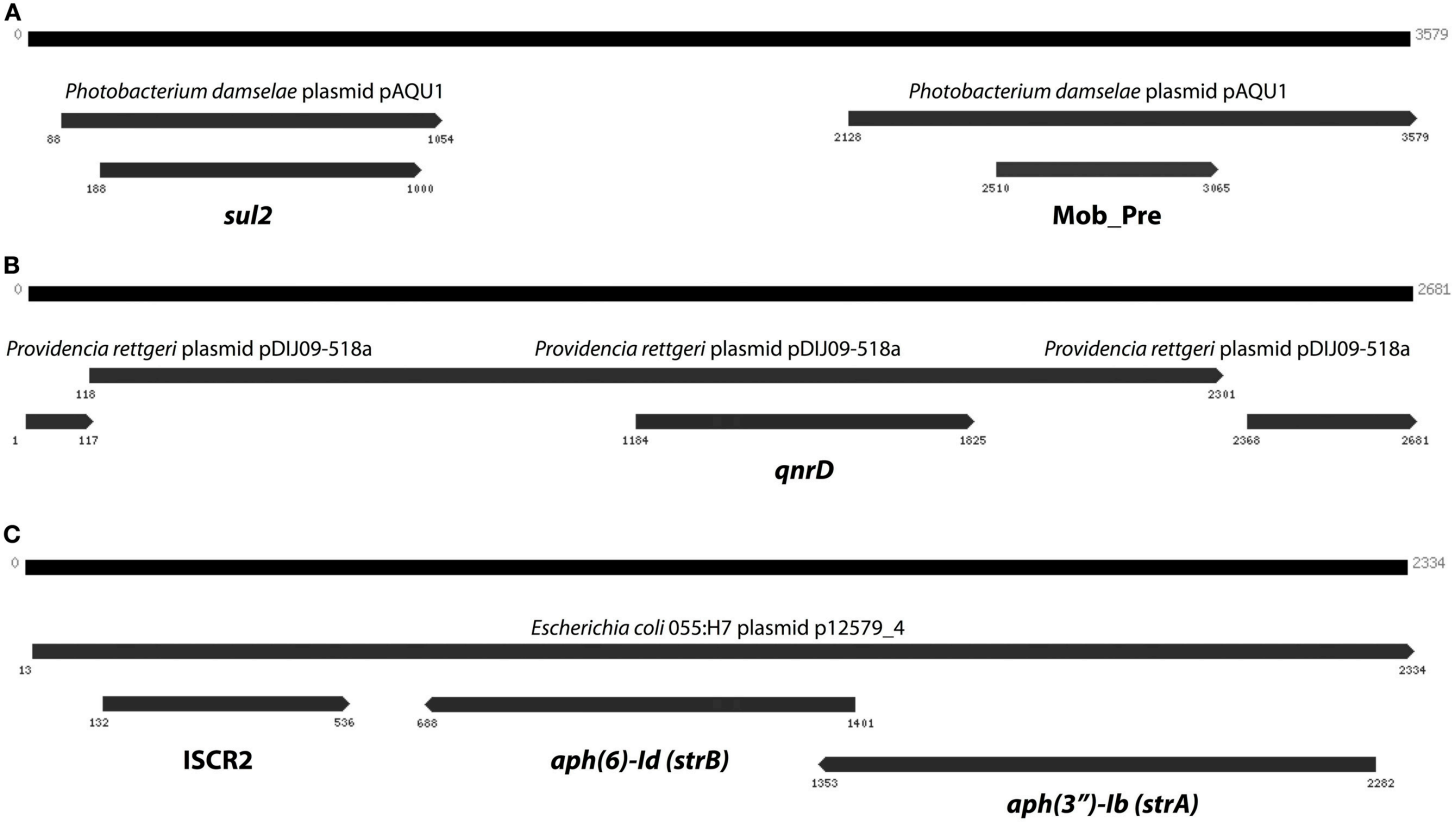
**The three longest contigs produced by targeted assembly of reads matching resistance genes**. **(A)** The *sul2* gene on a contig explaining 14% of the *sul2* abundance in the Indian lake. **(B)** A contig containing *qnrD* with 96% identity to a *Providencia rettgeri* plasmid. **(C)** A third contig containing the *aph(3″)-Ib* and *aph(6)-Id* streptomycin resistance genes (see Figure 5), this time in another genetic context. Numbers indicate the positions on the contig.

The curious absence of any long contigs containing the two most abundant resistance genes (*sul2* and *qnrD*), which instead were fragmented on several smaller contigs with lengths between 74 and 190 bp, suggests that those extremely common resistance genes could be present in multiple contexts and variants, and thereby cause Velvet to perform sub-optimally. We therefore devised a scheme in which Vmatch and Trinity were used to iteratively construct contigs from reads associated with resistance genes. This way, we were able to assemble a 3579 bp contig containing *sul2*, a 2681 bp contig containing *qnrD*, as well as a 2334 bp long contig carrying the *aph(3′)-Ib* (*strA*) and *aph(6)-Id* (*strB*) genes along with an ISCR2 mobile element (Figure 6).

In the Indian lake, 26 different plasmids present in the RefSeq plasmid genome database were recovered, of which 11 had a mean coverage of more than 100 (Table 3). In contrast, no complete plasmids were detected in the Swedish lake. Two of the recovered plasmids from the Indian lake contained fluoroquinolone resistance genes (*qnrD* and *qnrS2*). In addition, we could assemble 21 new putative small plasmids from the sequences from the Indian lake (Figure 4; Table 4). However, none of those contigs contained any known antibiotic resistance genes.

**Table 3:** **Known plasmids recovered from the Indian lake**.

**Plasmid accession**	**Plasmid name**	**Organism**	**Length (bp)**	**% GC content**	**Mean coverage**	**% covered**	**Associated resistance genes**
gi|514419625|ref|NC_021523.1|	pCGB40	*Escherichia coli strain* CGB40	4269	47.1	1890.8	100.0	*qnrD*
gi|431929441|ref|NC_019939.1|	pPSEST03	*Pseudomonas stutzeri* RCH2	2804	61.8	927.8	100.0	
gi|270208707|ref|NC_013548.1|	pRPSZY	*Rhodopseudomonas palustris*	2306	48.0	703.0	100.0	
gi|288551640|ref|NC_013780.1|	pAH3680	*Aeromonas hydrophila*	3680	55.8	579.4	97.1	
gi|507579660|ref|NC_021293.1|	pCSA2	*Cronobacter sakazakii* strain ATCC 29544	5103	55.0	397.3	99.2	
gi|189332428|ref|NC_010796.1|	pRK10	*Serratia marcescens*	4241	52.7	294.7	99.2	
gi|449306421|ref|NC_020262.1|	pSP291-3	*Cronobacter sakazakii* Sp291	4422	54.0	292.9	94.7	
gi|255929160|ref|NC_013090.1|	pLK39	*Endophytic bacterium* LOB-0*7*	4029	55.4	252.6	100.0	
gi|410688106|ref|NC_019242.1|	pS51B	*Enterobacter cloacae*	4854	52.4	241.7	99.0	
gi|209947505|ref|NC_011404.1|	pEC01	*Enterobacter cloacae*	5002	51.8	167.7	90.5	
gi|410654312|ref|NC_019132.1|	pSH1148_4.8	*Salmonella enterica* subsp. *enterica*	4775	53.3	111.5	96.4	
gi|435855463|ref|NC_019988.1|	pB1019	*Klebsiella pneumoniae* strain BB1088	5225	49.7	96.0	100.0	
gi|110666895|ref|NC_008246.1|	pYAN-1	*Sphingobium yanoikuyae*	5182	62.0	92.1	91.2	
gi|380083665|ref|NC_016979.1|	pUUH239.1	*Klebsiella pneumoniae*	5247	49.2	84.0	100.0	
gi|325168277|ref|NC_015182.1|	pACMV8	*Acidiphilium multivorum* AIU301	1729	60.9	57.0	100.0	
gi|10956807|ref|NC_002175.1|	pMTS1	*Methylophaga thalassica* strain S1	1995	42.5	54.6	98.1	
gi|410688217|ref|NC_019262.1|	pAHH04	*Aeromonas hydrophila*	7191	59.9	45.7	95.3	*qnrS2*
gi|389842917|ref|NC_017935.1|	pTHEBA.01	*Mesotoga prima* MesG1.Ag.4.2	1724	41.6	37.6	100.0	
gi|216996016|ref|NC_011640.1|	pKpn114	*Klebsiella pneumoniae*	4211	41.7	36.8	97.7	
gi|410609808|ref|NC_019092.1|	pLST424C-10	*Escherichia coli*	8848	49.1	36.1	97.4	
gi|194442198|ref|NC_011079.1|	pSL254_3	*Salmonella enterica* subsp*. enterica*	3605	43.0	30.5	100.0	
gi|410609294|ref|NC_019068.1|	pKST21	*Escherichia coli*	1460	51.0	13.2	98.0	
gi|209947511|ref|NC_011405.1|	pIGRK	*Klebsiella pneumoniae*	2348	33.4	12.5	100.0	
gi|410108997|ref|NC_019014.1|	pAAk1	*Aeromonas aquariorum* AAK1	4161	54.6	7.0	95.1	
gi|387615182|ref|NC_017723.1|	p58	*Escherichia coli* ETEC H10407	5800	48.9	6.9	90.4	
gi|189009159|ref|NC_010722.1|	pMM1	*Pseudomonas aeruginosa*	2140	45.8	4.4	92.6	

**Table 4:** **Novel plasmids retrieved from the Indian lake**.

**Plasmid contig**	**Length (bp)**	**% GC content**	**Avg coverage**.	**% covered**
lake_2007_contig_201	3295	58.1	89.7	100.0
lake_2007_contig_1230	4784	59.8	23.3	100.0
lake_2007_contig_30733	3975	48.9	16.1	100.0
lake_2007_contig_6114	5252	58.2	15.9	100.0
Peacat_contig_45232	5164	60.4	15.8	100.0
lake_2007_contig_5550	4525	51.0	14.3	100.0
lake_2007_contig_12425	5024	51.2	14.2	99.7
lake_2007_contig_4528	5356	44.8	11.3	100.0
lake_2007_contig_127348	3660	62.4	9.6	100.0
Peacat_contig_40251	4791	47.1	9.6	100.0
lake_2007_contig_19565	4418	55.6	9.2	99.4
lake_2007_contig_3701	5828	48.1	8.9	100.0
lake_2007_contig_10491	6072	46.8	7.7	99.3
lake_2007_contig_57444	3019	46.6	7.7	100.0
lake_2007_contig_42990	3153	36.6	7.6	100.0
lake_2007_contig_2219	7768	39.6	6.3	99.8
lake_2007_contig_187761	6203	32.2	5.8	100.0
lake_2007_contig_6987	4400	35.8	5.6	100.0
Peacat_contig_24026	3122	64.9	5.5	96.7
lake_2007_contig_12553	4616	44.0	5.5	100.0
lake_2007_contig_22993	3577	40.8	5.5	99.6

In total, 7310 and 8841 read pairs were identified as SSU sequences in the Indian and Swedish lake, respectively. The taxonomic origin of those read pairs were analyzed at different levels (Figure S3), revealing that the taxonomic composition of the two lakes differed substantially. The Indian lake was dominated by the Clostridia, Deltaproteobacteria, and Gammaproteobacteria classes, while the Swedish lake contained higher relative proportion of bacteria in the Betaproteobacteria and Planctomycetacia classes. The Swedish lake also had higher diversity at all investigated taxonomic levels (consistent with rarefaction analysis results; Figure S4), and also accomodated more unknown and/or unclassified bacterial 16S sequences. However, to us, the difference in diversity between the environments was surprisingly minor, given that the documented pollution of the Indian lake would be likely to pose a strong selection pressure on the community.

At the genus level, the Indian lake was—excluding unknown bacteria—dominated by *Pseudomonas, Sulfurimonas*, and *Spirochaeta* species. Additionally, species belonging to the Peptococcaceae, Helicobacteraceae, Desulforomonadaceae, and Desulfobacteraceae families and archaea of the Methanosarcinaceae family were also abundant. The Swedish lake, in contrast, was mostly preoccupied by unknown Proteobacterial genera (Figure S3).

## Discussion

In this work, we have used metagenomic sequence data to describe a high diversity of resistance genes in a highly antibiotic-polluted lake. By assembling the genetic context of the resistance genes, we could conclude that many are located on mobile genetic elements. We could detect high numbers of various genes involved in genetic mobility, in stark contrast with the investigated Swedish lake. Furthermore, we also identified 26 known and 21 novel complete plasmids in the Indian lake. The high abundances of both resistance factors and mobile genetic elements imply that polluted environments could serve as recruitment grounds for antibiotic resistance determinants that ultimately may end up in human pathogens. We also investigated the microbial diversity in the lake, and found that it contained a large number of different microbial taxa. This suggests that the acquisition of mobile resistance genes can contribute to the resilience of microbial communities in polluted environments.

While there are several possible explanations for the exceptional difference in relative abundance of resistance genes between the Indian and Swedish lakes, it should be noted that the total resistance gene abundance in the Indian lake is to our best knowledge only mirrored by one previously published metagenome, covering antibiotic-contaminated river sediments (Kristiansson et al., 2011). Obviously, there are a myriad of environmental and ecological differences between the two lakes. For example, the Indian lake is known to partially dry out in periods, while the Swedish lake becomes covered with ice during wintertime. Indeed, the average temperature in the Indian lake is higher throughout the year. The high abundances of several types of resistance genes in the Indian lake are not likely to be a mere reflection of the release of resistant bacteria from human or animal feces (Czekalski et al., 2012), as common gut bacteria such as *Prevotella, Bacteroides*, and *Ruminococcus* are only slightly more abundant in the Indian lake. Additionally, the levels of resistance genes in feces have been reported to be lower than the abundances we detected (Hu et al., 2013). That leaves two plausible explanations for the elevated resistance gene abundance. The first is that pollution of the lake has caused a selection pressure that promotes resistant bacteria on site. As the concentrations of fluoroquinolones reported in the lake water (Fick et al., 2009) approximately correspond to the clinical breakpoint for resistance, this is indeed a plausible explanation. Alternatively, resistant bacteria from antibiotic production have reached the lake via industrial wastewater. Both scenarios would be the result of disposal of waste from pharmaceutical production, but they cannot be distinguished with certainty from each other using the data of this study, and both may contribute to the observed resistance gene abundance.

### Abundance and diversity of resistance genes

Likely, pollution has had a dramatic impact on the resistance gene content of the Indian lake (Figure 1). The vast range of resistance gene classes detected suggests that some resistance factors could have been co-selected for under the selection pressure from different antibiotics and/or other factors. This could either be due to co-localization of those resistance factors with other resistance genes on the same mobile genetic element, co-conjugation—i.e., that the resistance factors are located on separate plasmids sharing parts of the conjugation system (Smillie et al., 2010), or caused by a general enrichment of mobile elements induced by strong selective pressure from chemical pollution. Fluoroquinolones are known to often co-select for resistance to several other types of antibiotics (Robicsek et al., 2006), but it is also plausible that other selective agents are present. Resistance genes were found to correspond to 0.316% of the reads in the Indian lake. As the DNA amplification procedure is not completely random, absolute gene abundances should be interpreted with some caution. The total estimated abundance is comparable to the levels found in contaminated river sediments (0.22%; Kristiansson et al., 2011), but far higher than what has been found in activated sludge (0.0054%; Yang et al., 2013), a Puget Sound marina (0.0017%; Port et al., 2012), municipal wastewater treatment plant effluent (0.012%; Port et al., 2012), or in sediment downstream from an effluent treatment plant in Sweden (0.02%; Kristiansson et al., 2011). In comparison, resistance genes in the Swedish lake was found to comprise 0.00003% of the total reads, which is lower than in the Puget Sound marina, but correspond well to data from other environments with little human impact (Port et al., 2012; Muziasari et al., 2014), where resistance genes are generally below the detection limit of previous studies. Consequently, if a lake with even less human activity would have been chosen for comparison, the levels of resistance genes may very well have been below the detection limit also of this study. Regrettably, comparing the number of different resistance gene types encountered between studies is not straightforward due to differences in read length distributions, sequencing errors, annotation strategies and sequencing effort. In this work, the sequencing depth would allow us to detect a resistance gene present in approximately one out of a thousand bacterial cells. Despite this limitation, we have found resistance genes to every major class of antibiotics in the Indian lake, along with a huge abundance of genetic elements promoting mobilization of DNA (Figures 2–4). In principle, this makes the lake a melting pot for antibiotic resistance, where genes that have not yet been mobilized are not unlikely to become in the future.

### Putative novel plasmids identified in the polluted lake

Given the large differences between the lakes in terms of abundances of plasmids, conjugation systems, and ISCR elements (Figure 4), it is reasonable to assume that the pollution have selected for increased horizontal gene transfer (Gillings, 2014). Similar environments have been shown to select for bacteria resistant to a broad range of antibiotics (Johnning et al., 2013; Marathe et al., 2013), and indeed we were able to assemble several small plasmids from the Indian lake, known as well as unknown (Tables 3, 4). Of the 26 known plasmids recovered, only two contained described resistance genes (*qnrD* and *qnrS2*). While most of these known plasmids have been isolated from human gut bacteria, the abundances of such genera were only slightly higher in the Indian lake compared to the Swedish. Thus, the presence of these plasmids are unlikely to be due to contamination with human feces in the Indian lake, although that may explain a minor portion of their abundance, or provide a possible source of those plasmids. There were furthermore no described resistance genes among the 21 putative novel plasmids assembled, indicating that the majority of the known resistance genes are located on larger plasmids, or in such a large number of genetic contexts that assigning them to a single genetic element would be nearly impossible by computational means. Sequencing protocols enabling longer read lengths would greatly aid such assemblies. Although the 21 novel plasmids identified did not carry any known resistance genes, they contained several unidentified open read frames (ORFs) that did not match to any Pfam family, even with permissive cutoffs. Such ORFs may encode proteins that are important for survival under the possibly variable and harsh environmental conditions that the Indian lake presents microbes with. Further evaluation of these novel plasmids using experimental methods would therefore be very interesting.

In total numbers, known resistance genes encompassed more than 0.3% of the reads obtained from the Indian lake, with the *sul2* and *qnrD* genes constituting 0.27% of the total reads. Likely, these genes would be present in more than one context; e.g., the recruitment of reads to the pCGB40 plasmid containing a *qnrD* gene, explains less than 10% of the total abundance of *qnrD* in the Indian lake. Since neither *sul2* nor *qnrD* showed up in any of the longer contigs assembled using Velvet, it seems that high abundance might be detrimental to assembly using standard means, rather than advantageous. As shown by our custom iterative assembly approach it can still be possible to assemble high-abundance, but greatly complicated, regions from metagenomic data using specially adapted means (Figure 6). This way, we were able to see that about 14% of the *sul2* reads were associated with a previously unknown genetic element, likely part of a plasmid, of which 79% of the sequence had 99% identity to the *Photobacterium damselae* plasmid pAQU1. Similarly, around 20% of the reads matching to *qnrD* could be explained by a contig assembled using this approach, with 96% identity to a plasmid from *Providencia rettgeri* (pDIJ09-518a). While these two assembled contigs are reassuring, they still only explain a minor portion of all the reads mapping to *sul2* and *qnrD*. Consequently, there seem to be even more variants, which might require even more sophisticated assembly algorithms to be untangled from the vast amount of reads corresponding to these two resistance genes.

### Increased general genetic mobility

Apart from the massive amount of resistance genes found in the Indian lake compared to the Swedish one, the immense difference in abundance of factors related to horizontal gene transfer between the two lakes was, by far, the most striking feature in terms of functional differences (Figures 2–4 and Figure S1; Tables S3, S4). Considering the most abundant GO-annotations in the two lakes, the Indian lake stands out with 2.85% of its reads connected to genetic transfer (Figure S5). This process had higher abundance than e.g., carbohydrate and protein metabolic processes, underscoring how extreme the abundance of this gene class is in the Indian lake. These results are clearly echoed among the differentially distributed level 3 GO terms in the samples, both based on Pfam families (Figure 2A), and TIGRFAM families (Figure 2B). The Indian lake showed large abundances of genes involved in genetic transfer, conjugation, transposition as well as plasmid maintenance in both Pfam and TIGRFAM results. It has been suggested that human activities that cause pollution with, e.g., pharmaceuticals, in the long run might influence the evolutionary rate of microorganisms (Gillings and Stokes, 2012). For example, *Streptococcus* species have been shown to become more susceptible to acquiring foreign DNA by transformation under antibiotic exposure (Prudhomme et al., 2006). This may, at least partially, be attributed to the general bacterial SOS response (Beaber et al., 2004). Our observation of a multitude of genes involved in horizontal gene transfer indeed supports this hypothesis.

The contamination of the Indian lake was not only reflected among antibiotic resistance genes, but also in the more general functionality of the bacterial community. Processes such as ion transport, arsenic detoxification, and sulfur metabolism were highly prevalent in the Indian sample, along with more general drug response and the broad-encompassing category of “response to stimulus.” Additionally, macromolecule metabolism also seems to be remarkably abundant in the Indian lake. The general detoxification gene content in the Indian lake is exemplified by findings of the Pfam profiles representing the copper resistance genes *CopB* (3.64 per million reads) and *CopC* (0.71 per million reads), the arsenic resistance gene *ArsC* (34.55 per million reads), the cadmium transporter *Cad* (0.58 per million reads), and the multidrug efflux family Multi_Drug_Res (66.62 per million reads; Table S3), as well as the TIGRFAM family *arsC* (arsenate reductase; 28.88 per million reads), and selenium and tellurium detoxification proteins belonging to the TMPT_Se_Te family (1.41 per million reads; Table S4). While one should be cautious about drawing conclusions from these data alone, they comprise a suggestion either for co-selection of antibiotic resistance genes and/or mobile elements with a range of detoxification genes; or for a strong general selection pressure by toxicants in the Indian lake. The latter scenario would not be unlikely due to discharge of industrial waste into the lake. Regardless of which, promotion of a broad range of resistance factors toward metals and other toxicants could also promote selection for antibiotic resistance in an environment where both mobile genetic elements and antibiotic resistance genes are prevalent (Pal et al., 2014).

### Community functions are reflected in taxonomy

From a taxonomic perspective, an interesting observation of this study is that the number of eukaryotes seems to differ dramatically between the two lakes. The Swedish lake harbored 2.73% eukaryote 18S rRNA sequences, while we only found 0.01% in the Indian lake. The same pattern was repeated for chloroplast and mitochondrial 16S rRNA (Figure S3). Archaea are often associated with extreme environments (Pikuta et al., 2007), but the overall abundance of archaeal species was lower in the polluted lake, although Euryarchaeota were more common. Moreover, the selection pressures in the Indian lake seem to promote known species and genera of bacteria to a larger extent than in the Swedish one, which consistently had higher number of unknown and unclassifiable taxonomic entries. Furthermore, the overall diversity also seems to be slightly lower in the Indian lake (Figure S4), as would be expected as a result of strong selection pressure (Banks et al., 2013). However, the difference was rather small, showing that a vast range of species actually do survive the comparably high levels of antibiotics and other chemicals present in the Indian lake (Fick et al., 2009). This is an interesting finding, as that indicates that a plethora of species are either intrinsically resistant, or have acquired resistance, and are therefore able to survive and reproduce in the contaminated lake.

The overall composition of species in the Indian lake showed high numbers of proteobacteria and firmicutes. Within proteobacteria, a large part of the abundance could be attributed to sulfate reducing bacteria such as Desulfobacteraceae, Desulfomicrobiaceae, and Desulfuromonadaceae. We also found large quantities of iron reducers in the Deferribacteraceae family, suggesting that both sulfur and iron compounds are important in the element cycling in the sediment community of the Indian lake. Additionally, we found large numbers of Clostridia, a class of obligate anaerobes, representing a strong indication that this environment might be very oxygen-poor. Clostridia and many other Firmicutes are able to form endospores, ensuring their survival in harsh conditions, such as drought. Since we found protein families related to spore formation in the Indian lake, but not in the Swedish lake (Tables S5, S6), the presence of microorganisms capable of such transformations is not surprising. Moreover, the Indian lake is known to be largely dried out in periods, meaning that spore formation might not only be a survival factor related to chemical stressors, but also to physical stress.

### Mitigating risks for spread and emergence of resistance factors

Given the broad range and relatively large abundance of known resistance genes we could identify in the Indian lake, it would be surprising not to find as yet undiscovered resistance genes in this environment. Many antibiotic resistance genes encountered in clinical settings are thought to originate from environmental bacteria (Forsberg et al., 2012; Wright, 2012; Finley et al., 2013; Gaze et al., 2013), where they probably have been present for millions of years (D'Costa et al., 2011). It would be astonishing if this gene-flow suddenly stopped, and novel antibiotic substances we discover would not face horizontally-transferred resistance development. However, the more likely scenario is that new resistance genes await us down the line, as has happened with e.g., fluoroquinolones and carbapenems (Lupo et al., 2012). Such novel resistance genes can be hard to discover using bioinformatic approaches, although a great deal can be achieved by analysis of homologous sequences (Boulund et al., 2012; Flach et al., 2013). Instead, studies of genetic material from environmental communities inserted into laboratory strains subjected to selection pressures, so called functional metagenomics (Handelsman et al., 1998), will be instrumental in detecting such completely novel resistance genes. Functional metagenomics has been previously applied to detect e.g., novel beta-lactamases (Allen et al., 2009) and chloramphenicol resistance genes (Lang et al., 2010). Such large-scale screenings of heavily contaminated environments similar to the Indian lake could provide us with important insights into the range and nature of resistance genes that we might face in the clinic in the future.

An important aspect of research on environmental antibiotic resistance is to track down potential sources of antibiotic contamination, and particularly to assess in which arenas transfer of resistance genes from environmental bacteria into pathogens is more likely to occur (Pruden et al., 2013). Often, milieus such as wastewater treatment plants have been proposed as such potential high-risk environments (Rizzo et al., 2013). However, the findings of this work and previous studies of environments contaminated by pharmaceutical production waste (Kristiansson et al., 2011) suggest that much more effort is needed in unearthing risks related to pollution from production facilities. Particularly, risks are high in settings where humans and/or animals interact with polluted water, soil or other materials. In such situations, there is an obvious risk that resistance genes selected for in a polluted environment might spread into the human or animal gut microflora, and thereafter propagate into pathogens or pathogenic opportunists. Consequently, more studies are needed on resistance genes and their mobility in highly polluted areas as well as in their neighboring milieu, and further on in the human microbiome of their inhabitants. It is of high importance to identify such pollution sources, and to inform management (Pruden et al., 2013). Furthermore, we also need to understand how resistance plasmids evolve and spread, and which factors can assist in such processes. Metagenomics can play a significant role in identifying settings of particularly high risk, and thereby suggest where to start future mitigation efforts.

## Author contributions

D. G. Joakim Larsson, Erik Kristiansson, and Jerker Fick designed the study. Jerker Fick was responsible for collecting the samples. Johan Bengtsson-Palme and Fredrik Boulund performed the bioinformatics analysis. Johan Bengtsson-Palme, Fredrik Boulund, Erik Kristiansson, and D. G. Joakim Larsson interpreted the results. Johan Bengtsson-Palme, Erik Kristiansson, and D. G. Joakim Larsson drafted the manuscript. All authors read and approved the final manuscript.

### Conflict of interest statement

The authors declare that the research was conducted in the absence of any commercial or financial relationships that could be construed as a potential conflict of interest.
