# Supplementary material for: Shotgun metagenomics reveals a wide array of antibiotic resistance genes and mobile elements in a polluted lake in India
Source: Front Microbiol. 2014 Dec 2;5:648. doi: 10.3389/fmicb.2014.00648 (PMC4251439; doi:10.3389/fmicb.2014.00648)

Figure S1. Pfam families related to mobility, and their abundance in the Indian (black) and Swedish (white) lake. Only families with a coverage of at least 1 read per million sequences are shown, for the full list of investigated Pfam families related to genetic mobility, see Table S7.

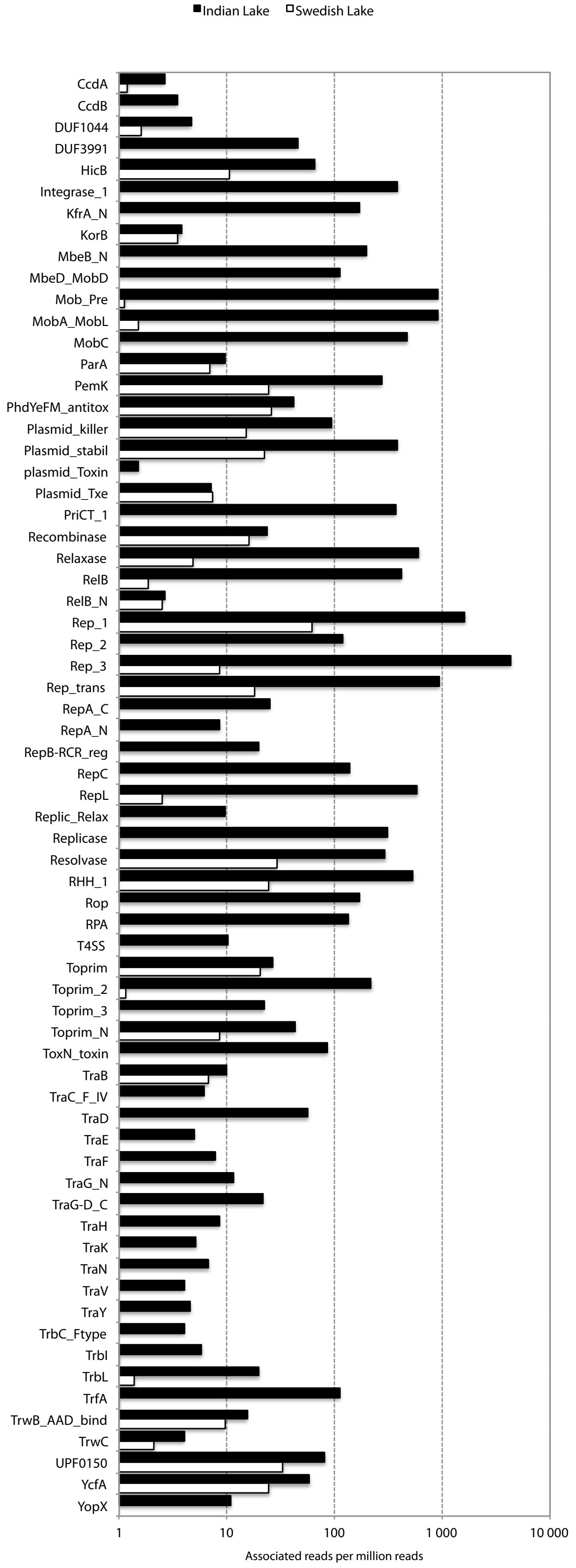

Supplement: Supplementary file 10 [file Image1.PDF]
