# Supplementary material for: Shotgun metagenomics reveals a wide array of antibiotic resistance genes and mobile elements in a polluted lake in India
Source: Front Microbiol. 2014 Dec 2;5:648. doi: 10.3389/fmicb.2014.00648 (PMC4251439; doi:10.3389/fmicb.2014.00648)

Figure S2. Different types of *intI* integrases and ISCR elements identified in the Swedish (white) and Indian (black) lake.

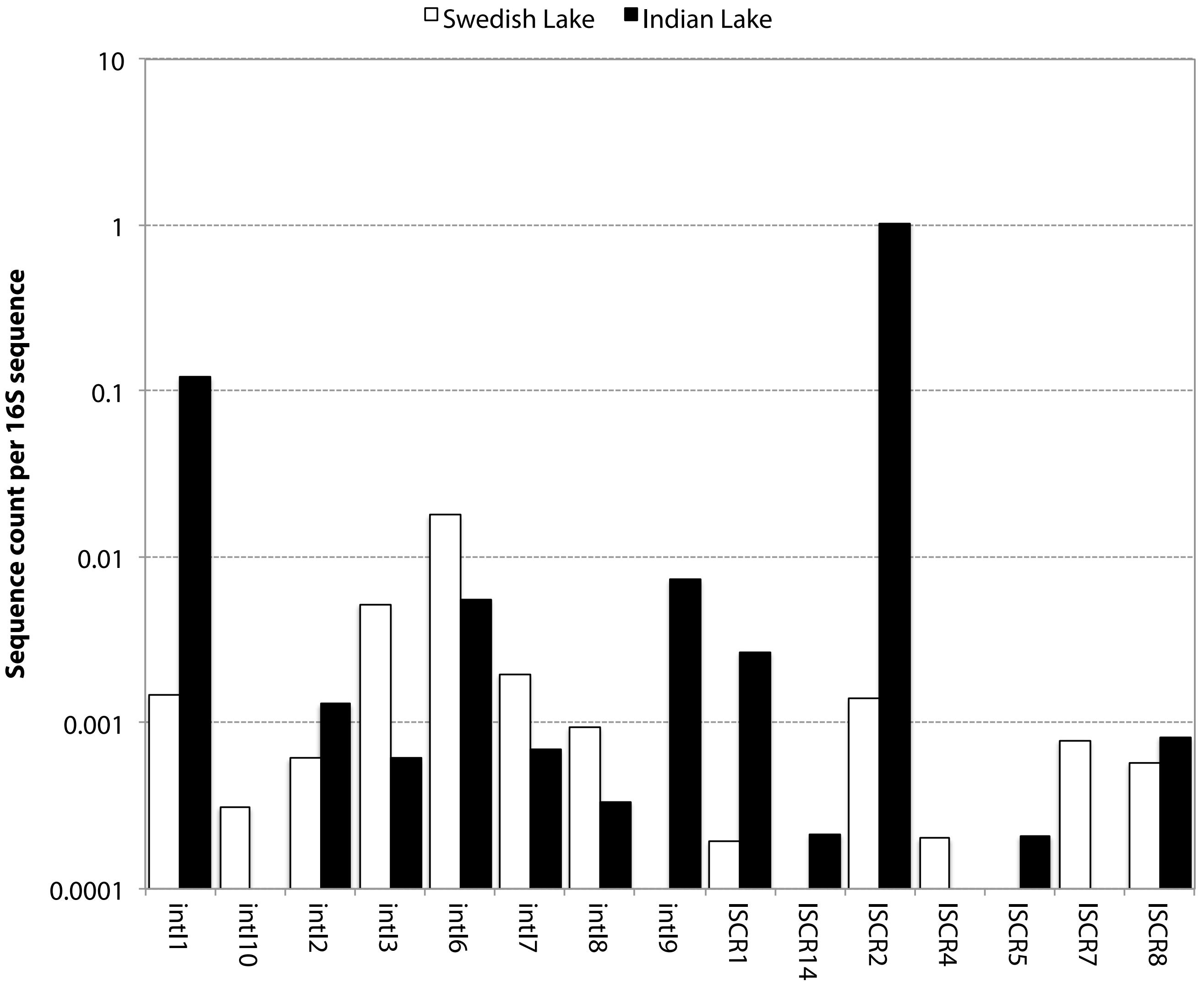

Supplement: Supplementary file 11 [file Image2.PDF]
