# Supplementary material for: Shotgun metagenomics reveals a wide array of antibiotic resistance genes and mobile elements in a polluted lake in India
Source: Front Microbiol. 2014 Dec 2;5:648. doi: 10.3389/fmicb.2014.00648 (PMC4251439; doi:10.3389/fmicb.2014.00648)

Figure S3. Taxonomy of the Indian and Swedish lakes. (a) Proportion of SSU sequences annotated as Archaea, Bacteria, Eukaryota, Chloroplast, Mitochondria or Unknown by Metaxa. (b) Relative abundance of the most prevalent phyla (≥ 1% in at least one of the lakes) in the two lakes. (c) Relative abundance of taxonomic families constituting more than 1% of the SSU rRNAs in at least one of the lakes.

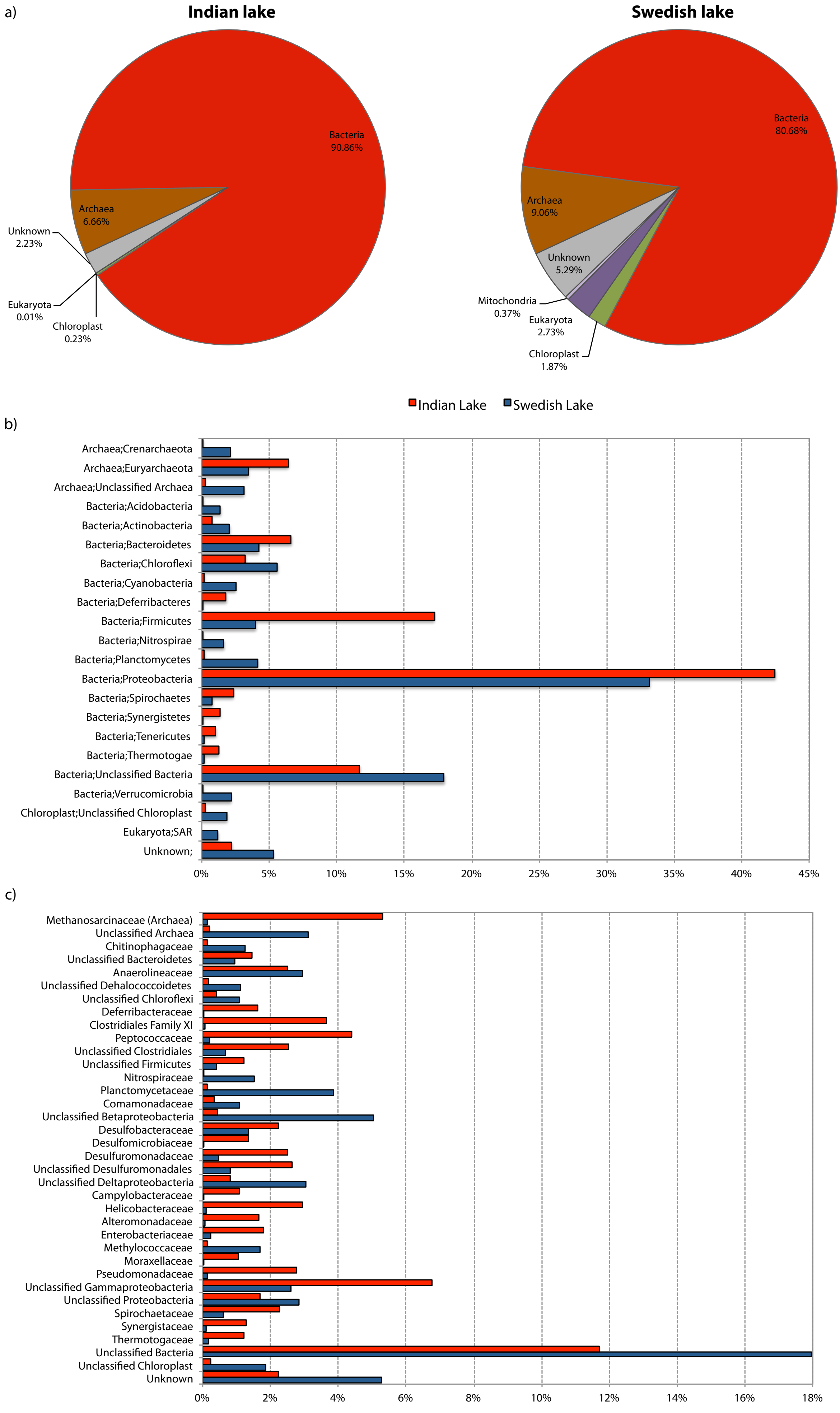

Supplement: Supplementary file 12 [file Image3.PDF]
