# Supplementary material for: Shotgun metagenomics reveals a wide array of antibiotic resistance genes and mobile elements in a polluted lake in India
Source: Front Microbiol. 2014 Dec 2;5:648. doi: 10.3389/fmicb.2014.00648 (PMC4251439; doi:10.3389/fmicb.2014.00648)

Figure S4. Species accumulation curves generated using Megraft and Vegan, from bacterial 16S rRNA fragments extracted by Metaxa, from the Indian (red) and Swedish (blue) lakes. These curves indicate that the Swedish lake is slightly more diverse in terms of taxonomy, than the Indian one.

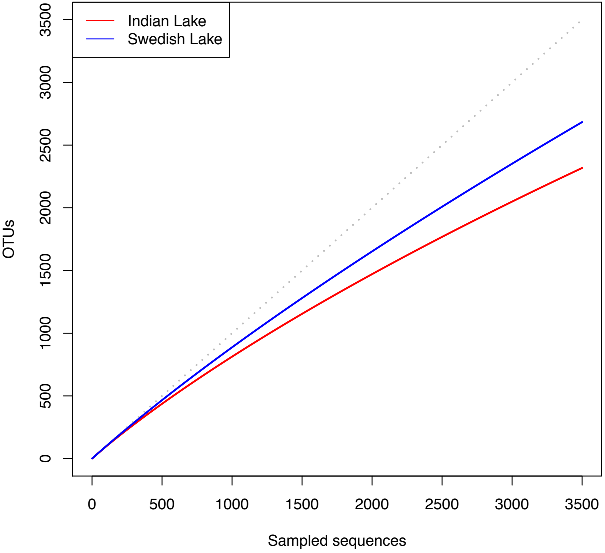

Supplement: Supplementary file 13 [file Image4.PDF]
