# Supplementary material for: Shotgun metagenomics reveals a wide array of antibiotic resistance genes and mobile elements in a polluted lake in India
Source: Front Microbiol. 2014 Dec 2;5:648. doi: 10.3389/fmicb.2014.00648 (PMC4251439; doi:10.3389/fmicb.2014.00648)

Figure S5. Level 3 GO-terms associated with biological processes representing more than 1.0% of the Pfam families encountered in the Indian (left) and Swedish (right) lakes. All GO-terms less abundant than 1.0% has been grouped into the "Other" category.

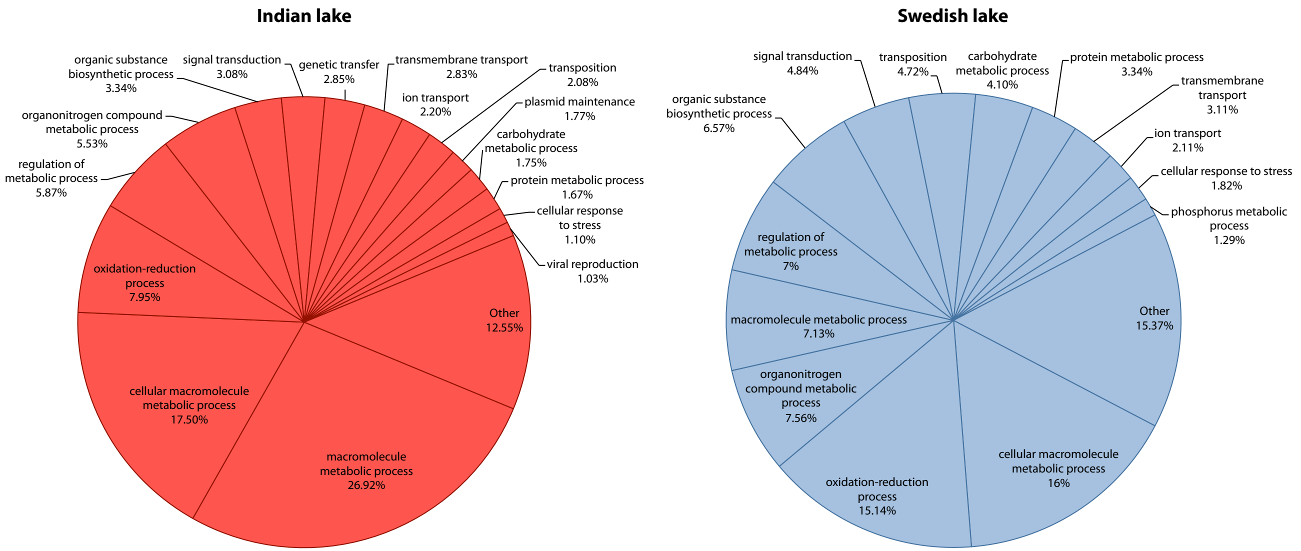

Supplement: Supplementary file 14 [file Image5.PDF]
